# Practice Testing Facilitates Forward Navigation but Undermines Backward Navigation During Map Learning

**DOI:** 10.3390/jintelligence13040049

**Published:** 2025-04-15

**Authors:** Shaohang Liu, Chunliang Yang

**Affiliations:** 1 Institute of Developmental Psychology, Faculty of Psychology, Beijing Normal University, Beijing 100875, China; 11132022025@bnu.edu.cn; 2 Beijing Key Laboratory of Applied Experimental Psychology, National Demonstration Center for Experimental Psychology Education, Beijing Normal University, Beijing 100875, China

**Keywords:** testing effect, map learning, forward navigation, backward navigation, peripheral information

## Abstract

Practice testing (i.e., retrieval practice) has been established as a powerful learning strategy by comparison with many others, such as restudying. The current study explores whether practice testing can boost learning of map routes. Experiment 1 demonstrated that, by comparison with restudying, testing enhanced forward navigation and facilitated memory for peripheral information along the route. Experiment 2 examined the testing effect on backward navigation by asking participants to navigate from the endpoint to the start point in the final recall test. The results showed a negative testing effect: testing produced poorer backward navigation performance by comparison with restudying. Experiment 3 demonstrated that showing participants the tracing of the cursor during the retrieval practice phase eliminated the negative testing effect on backward navigation. Overall, the documented findings suggest that retrieval practice can facilitate forward navigation but impair backward navigation when the navigation task requires reorganization and mental rotation of the learned routes.

## 1. Introduction

Practice testing has been repeatedly established as a more efficient study strategy in consolidating long-term retention in comparison to many others, such as restudying, note-taking, and so on. This phenomenon is known as the *testing effect*, *retrieval practice effect*, or *test-enhanced learning* (for reviews, see Roediger and Karpicke 2006; Rowland 2014; Shanks et al. 2023; Yang et al. 2021). Test-enhanced learning has been observed with a range of learning materials, including verbal materials, such as word pairs, foreign-translation word pairs, science facts and processes, and nonverbal materials, such as Chinese characters (Kang 2010; participants without Chinese knowledge), maps (Carpenter and Pashler 2007), and human faces (Carpenter and DeLosh 2005).

Test-enhanced learning has not only been observed in classroom settings (e.g., McDaniel et al. 2007 for a review, see Yang et al. 2021) but also in other daily or professional circumstances. A typical example is map learning, that is, memorizing the information on a map (e.g., route, landmark coordinate, etc.) and utilizing the map information to solve real problems (e.g., navigation). On some occasions, the map users (e.g., troops, police, or outdoor explorers) do not have access to the map all the time so, sometimes, they have to rely on their memory of the map to complete the journey. Thus, it is necessary to explore optimal study strategies for facilitating map learning. Although map learning is ubiquitous in daily life, very few studies have explored whether practice testing can facilitate map learning. More importantly, the most common circumstance of memorizing a route from one place to another has never been explored (see below for details). To fill these important gaps, the present study explored whether test-enhanced learning persists in a range of aspects of map learning, including memorizing a route, back-tracing the route, and memorizing the peripheral information of the route. Below, we briefly review related literature.

## 2. Testing Effect on Map Learning

Even though many studies have explored the testing effect on visuospatial memory, to our knowledge, only three studies so far have explored the effect of testing on map learning. A pioneering study was conducted by Carpenter and Pashler (2007), in which participants learned a map presenting rivers, roads, and bridges. During the review phase, participants in the restudy group reviewed the map, whereas those in the test group recalled the item that was randomly omitted and then pressed the button to make the missing part reappear. The results showed that retrieval practice produced superior recall of the map when participants were asked to draw the map after 30 min. Another study by Rohrer et al. (2010) asked participants to memorize the position of cities on a map. In the restudy condition, participants restudied the map after the initial learning phase. In the test condition, after the initial learning phase, participants were asked to put a given city name in the correct position and then received corrective feedback. In the final test, participants were cued by positions on the map and recall the city name corresponding to each position. Final test performance was substantially better in the test than in the restudy condition, reflecting a testing effect on map learning. The third study was conducted by Carpenter and Kelly (2012), in which participants were instructed to memorize the three-dimensional layout of objects. After initial learning, participants either restudied the layout or imagined standing at the position of one object facing another object and then reported the direction of a third object. In the final test, participants produced significantly better memory for the layout in the test than in the restudy condition. Overall, all three studies consistently demonstrated that testing is a more powerful strategy in facilitating map learning than restudying (Carpenter and Kelly 2012; Carpenter and Pashler 2007; Rohrer et al. 2010).

A possible explanation for the testing effect on map learning is the *transfer-appropriate processing theory* (Morris et al. 1977), which proposes that, compared with restudying, practice test and the final test share a greater resemblance; hence, final test performance is better in the test than in the restudy condition. Another account, termed as the *episodic context theory*, asserts that the benefits of retrieval practice derive from updates of episodic contexts (Lehman et al. 2014; Whiffen and Karpicke 2017). Specifically, when participants recall a given item during the practice test, they will reinstate the episodic context where the item is initially encoded and link the retrieval context with the initial encoding context. Therefore, when participants are prompted to recall the item in the final test, both the context of initial encoding and that of practice retrieval jointly aid its successful retrieval. By contrast, a restudied item is only associated with encoding context, without retrieval context (Karpicke et al. 2014; Lehman et al. 2014).

## 3. Rationale of the Present Study

The daily use of map information not only involves retrieving object locations but also integration of the location information to accomplish practical missions, including indicating relative positions, navigating from one place to another, searching for the target positions on a given map, and so on. Previous studies have examined the transfer effect of testing on map learning by showing that the testing effect survived when the format of the practice test and that of the final test were different. For example, in Rohrer et al. (2010), the final test included test questions that were either the same, similar (i.e., filling the blank on a map according to a city name list), or completely different from those in the practice test (i.e., recalling the name of a city located between another two cities). The result is that the testing effect is transferable to similar and different questions. However, previous studies overlooked the occasion where map information is most frequently used. In daily life circumstances, the purpose of learning a map is not barely knowing Point A is to the east of Point B or Point C is at the top-left corner of the map. Instead, the most common situation is to memorize the route from Point A to Point B. In this fashion, learners not only need to memorize the association between a given location and its label (as was conducted by previous studies) but also need to know how to arrive at a position from the current position. Additionally, it is also critical and common to memorize some peripheral information along the route (e.g., the location of a supermarket alongside the road).

In sum, while the testing effect has been extensively studied in various domains, its application to map learning, particularly in dynamic navigation tasks, remains underexplored. Previous research has primarily focused on static map features, such as item positions (Carpenter and Pashler 2007) and city names (Rohrer et al. 2010), but has not addressed the most common real-world scenario: memorizing and navigating a route. Furthermore, it is unclear whether retrieval practice enhances memory for peripheral information along the route or whether it facilitates backward navigation, which requires mental rotation and reorganization of the learned route. These gaps highlight the need for a more comprehensive investigation into the role of retrieval practice in map learning. Based on this, there are three main research questions explored in the present study. First, we aim to investigate whether test-enhanced learning exists in the most common map-using circumstance—memorizing a route on a map. Then, we test two types of transfer learning that are closely associated with daily map using: (a) Does practice retrieval of a route also boost memory of peripheral information along the route? (b) Does retrieval practice of forward navigation (i.e., navigation from the start point to the destination) also facilitate backward navigation (i.e., navigation from the destination back to the start point)?

## 4. Experiment 1: Forward Navigation

Experiment 1 was conducted to explore whether, by comparison with restudying, practice testing can more effectively enhance forward navigation performance. In addition, it also aimed to explore the transfer effect of testing on memory of peripheral information along the route.

### 4.1. Method

*Participants.* Given that map-route materials have never been employed in previous studies, we could not conduct sample size justification based on the effect sizes reported in previous studies. Instead, we adopted a medium effect size ($\eta_{p}^{2}$ = 0.06) as default, setting a statistical power of .95. A power analysis showed that the minimum sample size was 54 participants. We finally recruited 60 participants (35 female; *M*_age_ = 22.9, *SD* = 1.6) from Harbin Normal University. All participants signed an agreement to participate, reported normal or corrected-to-normal vision, were individually tested in a sound-proofed cubicle, and received monetary compensation. All participants signed the consent form and received CNY 25 as compensation. All experiments reported in the present study were approved by the Ethics Committee of Beijing Normal University, Faculty of Psychology.

**Figure 1:**
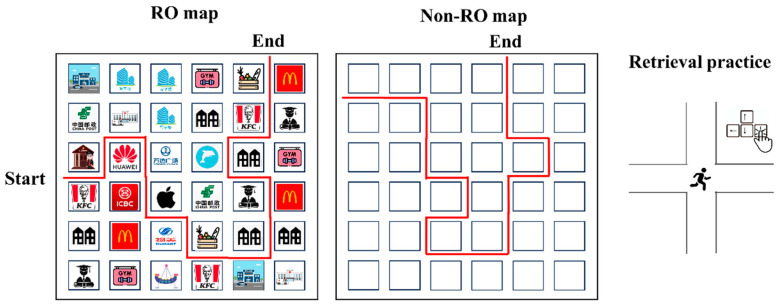
Procedure of Experiment 1.

*Materials.* Study stimuli were four maps, including two maps with reference objects (i.e., RO maps; see the left panel of Figure 1 for an example) and two maps without reference objects (no-RO maps; see the right panel of Figure 1 for an example). Each RO map consisted of 36 (=6 × 6) blocks and each block represented a landmark (e.g., a hospital). The icons of the landmarks were identifiable and familiar to participants. Participants could obtain the name of a given icon by clicking it. All the roads on the map were horizontal or vertical and a to-be-remembered route was flagged on the map. From the starting point to the destination, participants walked by 16 crosses, which means (during retrieval practice and final test) they needed to make 16 one-out-of-three decisions in total for each map. In the no-RO maps, no identifiable areas are shown on the map and each block is represented by an identical white square (see Figure 1). Each map was paired with an artificial name (e.g., *huaiyuan zhen*, representing a town named *huaiyuan*).

*Design and procedure.* Experiment 1 involved a 2 (study strategy: restudying vs. testing) ×2 (map type: RO map vs. no-RO map) within-subjects design. The experiment was programed by PsychoPy 2022.2.5 (Peirce 2007). Participants were tested individually using a desktop with a 27-inch screen, and they were seated approximately 50 cm from the screen.

The experiment consisted of three sessions, including initial study, practice (restudying or testing), and final test. In the initial study phase, participants viewed 2 RO (maps A and B) and 2 no-RO maps (maps C and D), with each map shown for 3 min for participants to study. The order of four maps was counterbalanced; the assignment of restudy/testing map was also counterbalanced. The map was presented at the center of the screen (size = 800 × 800 pixels). After initial learning, participants engaged in a 2 min distractor task in which they solved mathematical problems. Then, they either undertook a practice test or restudied the maps.

In the restudy condition, participants had 1 min to review the whole map, which was identical to what they carried out in the initial study phase. In the test condition, participants controlled a “cursor” at the starting point and followed the studied map to move the cursor from the starting point to the destination. They pressed the “left”, “right”, “up”, or “down” button to move the cursor to the next cross. If participants made a correct selection, the cursor proceeded to the next cross, with a “tick” mark popping up on the screen. If not, a “cross” mark was shown on the screen, the cursor stayed at the current position, and participants needed to make another attempt. Until they correctly selected the direction, the cursor proceeded to the next cross. This means that participants could obtain feedback for incorrect decisions. The total time for the retrieval practice was also 1 min. The program proceeded to the next session when time was up. The order of the practiced/restudy trials was counterbalanced.

After the practice phase, the first-day experiment finished and participants were dismissed. Twenty-four hours later, they returned to the lab and completed the final test. The final test had two parts. In the first part, participants completed a peripheral information test (PIT) by answering 10 questions related to each RO map, such as “*How many hospitals did you see on the map of huaiyuan zhen?*” and “*If you are in huaiyuan zhen and standing at north of court and facing north, which place can you see?*”. After finishing the PIT, participants completed a route test on four maps. The procedure of the route test was identical to that in the practical test, except that participants were not allowed to have additional attempts if they did not successfully recall the direction at a cross. Specifically, if participants made a wrong selection at a given cross, the cursor automatically jumped to the next cross. Performance of forward navigation was counted as the number of crosses at which participants selected the correct direction. The order of PIT and route test was counterbalanced across participants.

### 4.2. Results and Discussion

**Figure 2:**
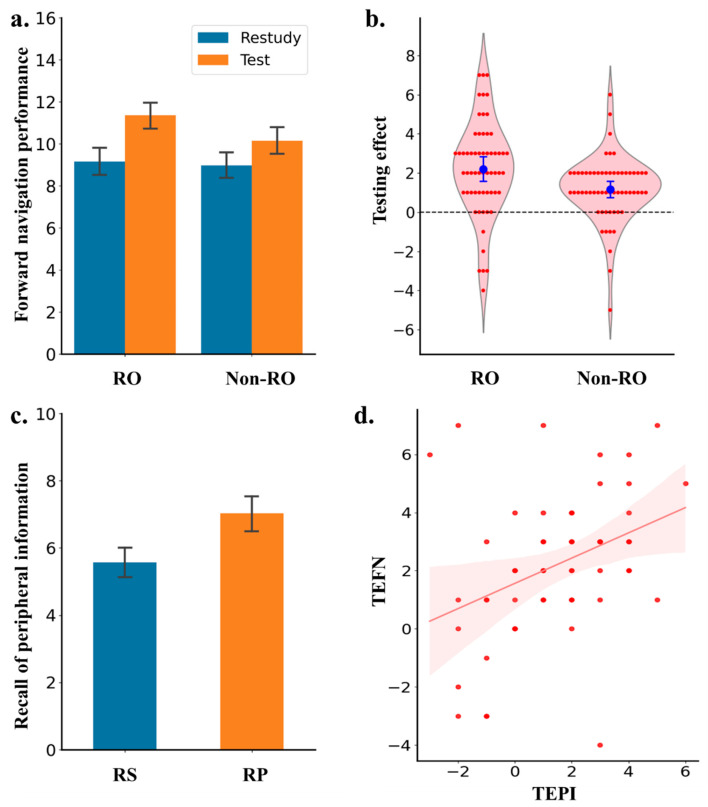
Results of Experiment 1. (**a**) Forward navigation performance for the route with referencing objects and without referencing objects. (**b**) The testing effect (i.e., the difference in performance between restudy and retrieval practice condition). (**c**) Performance in recalling peripheral information for restudy and retrieval practice condition. (**d**) The relationship between the testing effect on peripheral information and the testing effect on backward navigation. RO = referencing objects; Non-RO = non-referencing objects; RS = restudy; RP = retrieval practice; TEBN = testing effect on forward navigation; TEPI = testing effect on peripheral information. Error bars indicate 95% confidence interval.

Route memory performance (i.e., forward navigation performance) is visually depicted in Figure 2a as a function of study strategy and map type. A 2 × 2 analysis of variance (ANOVA), with study strategy (restudy vs. retrieval practice) and map type (RO map vs. non-RO map) as within-subjects independent variables, showed superior memory performance in the test compared to the restudy condition; difference = 1.68, *CI* = [1.32, 2.05], *F*(1,59) = 84.31, *p* < .001, η_p_^2^ = .59, *BF*_10_ > 1000. The main effect of map type was not statistically detectable; difference = 0.70, CI = [0.05, 0.61], *F*(1,59) = 2.89, *p* = .10, η_p_^2^ = .05, *BF*_10_ = 1.08. The interaction between the two factors was significant; *F*(1,59) = 6.89, *p* = .011, η_p_^2^ = .11, *BF*_10_ = 3.235. Specifically, as shown in Figure 2b, this interaction was mainly derived from the fact that the magnitude of test-enhanced learning was significantly larger in the OR map than in the no-RO map condition; difference = 1.217 95% CI [−0.015, 0.991], *t*(59) = 2.665, *p* = .004, *d* = 0.488, *BF*_10_ = 14.206. Furthermore, simple-effect analyses demonstrated that forward navigation performance was always better for tested than that for restudied maps in both the RO map condition, difference = 1.17 [0.74, 1.60], *t*(59) = 5.41, *p* < .001, *d* = 0.70, *BF*_10_ > 1000, and the no-RO map condition, difference = 2.20 [1.57, 2.83], *t*(59) = 7.02, *p* < .001, *d* = 0.91, *BF*_10_ > 1000. 

Besides facilitating route memory (i.e., measured as forward navigation performance), testing also enhanced memory of peripheral information along the route; difference = 1.47 95% CI [0.93, 2.01], *t*(59) = 3.98, *d* = 0.70, *BF*_10_ > 1000 (see Figure 2c). Additionally, there was a positive correlation between the testing effect on route memory (measured as the difference in forward navigation performance between tested and restudied maps) and the testing effect on the memory of peripheral information (measured as the difference in recall of peripheral information between tested and restudied maps), *r* = .38, *p* = .003, *BF*_10_ = 11.24 (see Figure 2d).

Finally, a within-subjects mediation analysis was performed, with study strategy (restudy vs. retrieval practice) as the independent variable, recall performance of peripheral information as the mediator, and route memory (i.e., forward navigation performance) as the dependent variable. The mediation analysis was performed by SPSS 25.0. (Montoya 2019), in which data of no-RO maps were excluded. The direct effect of the study strategy on route memory performance was significant; *c*’ = 1.60, 95% CI [0.87, 2.32], *p* < .001. More importantly, a bias-corrected bootstrap resampling analysis (with 5000 resamples) and normal theory tests indicated that the indirect effect of testing on forward navigation via its effect on memory of peripheral information was significant; *a* × *b* = 0.60, 95% CI [0.07, 1.26], *p* = .006.

Experiment 1 demonstrated that, by comparison with restudying, practice testing not only produced better route memory (as reflected by better forward navigation performance in the test than in the restudy condition) but also promoted transfer of map learning (as reflected by better recall performance of peripheral information in the test than in the restudy condition). Furthermore, the correlation and mediation results suggest that retrieval practice facilitated forward navigation partially through its enhancing effect on memory of peripheral information. Put differently, retrieval practice may induce superior encoding of peripheral information, which, in turn, contributes to the testing effect on forward navigation performance. This explanation can also help explain why the testing effect on RO maps was larger than the effect on no-RO maps.

## 5. Experiment 2: Backward Navigation

Different from Experiment 1, Experiment 2 investigated the transfer effect of practice testing on backward navigation (i.e., walking from the destination back to the starting point).

### 5.1. Method

*Participants.* Following Experiment 1, 60 participants were recruited from Harbin Normal University (38 female; *M*_age_ = 20.2, *SD* = 1.8). All participants signed the consent form and obtained CNY 25 as compensation.

*Materials, design, and procedure.* The materials, experimental design, and procedure were identical to those in Experiment 1, except that participants in Experiment 2 finally completed a backward navigation test. Specifically, in the final test, participants needed to move the cursor from the endpoint to the start point.

### 5.2. Results and Discussion

**Figure 3:**
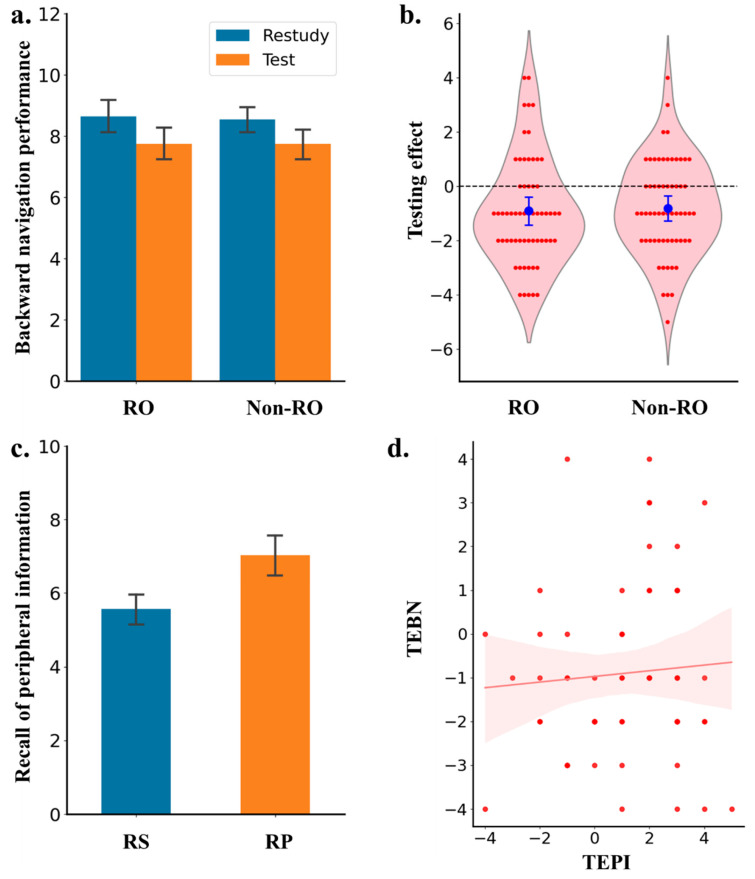
Results of Experiment 2. (**a**) Backward navigation performance for the routes with referencing objects and without referencing objects. (**b**) The testing effect (i.e., the difference in performance between restudy and retrieval practice condition) for RO and Non-RO condition. (**c**) Performance in recalling peripheral information for restudy and retrieval practice condition. (**d**) The relationship between the testing effect on peripheral information and the testing effect on backward navigation. RO = referencing objects; Non-RO = non-referencing objects; RS = restudy; RP = retrieval practice; TEBN = testing effect on backward navigation; TEPI = testing effect on peripheral information. Error bars indicate 95% confidence interval.

Route memory performance (i.e., backward navigation performance) is visually illustrated in Figure 3a,b as a function of study strategy and map type. Surprisingly, a 2 × 2 ANOVA showed a negative testing effect, with poorer backward navigation performance in the test than in the restudy condition; difference = −0.85, 95% CI = [−0.63, −024], *F*(1,59) = 24.09, *p* < .001, η_p_^2^ = 0.07, *BF*_10_ = 15.46. There was no main effect of map type, difference = 0.05, 95% CI [−0.31, 0.36], *F*(1,59) = 0.22, *p* = .88, η_p_^2^ = 2.32 × 10^−4^, *BF*_10_ = 0.24, nor the interaction between the two factors, *F*(1,59) = 0.09, *p* = .77, η_p_^2^ < .001, *BF*_10_ = 0.22.

In line with Experiment 1, testing produced significantly better recall of peripheral information than restudying; difference = 1.22, 95% CI = [0.25, 0.79], *t*(59) = 4.06, *p* < .001, *d* = 0.524, *BF*_10_ = 151.9, (see Figure 3c). However, unlike Experiment 1, the testing effect on backward navigation was not correlated with the effect on memory of peripheral information; *r* = .069, *p* = .598, *BF*_10_ = 0.185 (see Figure 3d).

Experiment 2 found a result pattern rather different from that in Experiment 1: practice testing of forward navigation impaired backward navigation performance in the final test and the mnemonic advantage of testing on memory of peripheral information contributed minimally to the negative testing effect on backward navigation performance. A possible explanation is that the key to complete backward navigation is a visuospatial representation of the route. Unlike the restudy condition in which participants could view the “complete visuospatial shape” of the route, practice testing broke down the route into different segments and so impaired visuospatial representation of the route.

**Figure 4:**
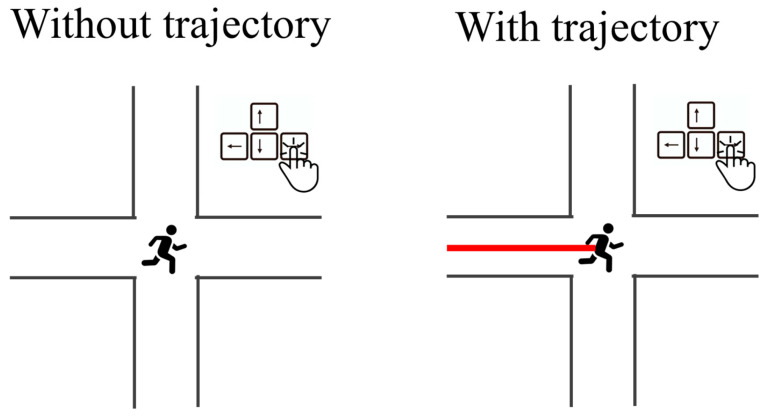
Retrieval practice without (Experiment 2) and with concurrent trajectory (Experiment 3).

Experiment 3 aimed to test this explanation by enhancing the visuospatial processing of the route during the retrieval practice session. Specifically, in Experiment 3, when participants moved the cursor during the retrieval practice session, the passing-by trajectory was concurrently presented on the screen (see Figure 4), which is expected to facilitate visuospatial representation of the route. We hypothesized that this manipulation would reduce (or even overturn) the negative effect of testing on backward navigation performance.

## 6. Experiment 3: Theoretical Explanation Test

As aforementioned, Experiment 3 was conducted to explore whether enhancing visuospatial representation of the route by showing passing-by trajectory can mitigate or even overturn the negative effect of testing on backward navigation performance.

### 6.1. Method

*Participants.* Following Experiments 1 and 2, 60 participants were recruited from Harbin Normal University. Four of them did not attend the delayed memory test and were therefore excluded from data analyses, leaving final data from 56 participants (40 female; *M*_age_ = 19.9, *SD* = 1.7). All participants signed an agreement to participate, reported normal or corrected-to-normal vision, were individually tested in a sound-proofed cubicle, and received monetary compensation.

*Materials.* The learning materials were three OR maps similar to those used in Experiment 1.

*Design and procedure.* Experiment 3 involved a within-subjects design (study strategy: restudying vs. forward practice vs. backward practice). Participants learned three RO maps in the initial learning phase in random order, with each map studied for 1 min. After initial learning, they engaged in a 2 min calculation task as a distractor. Then, participants successively restudied one map, took a practice test of forward navigation on one map, and took a practice test of backward navigation on the other map. The order of the three practice conditions was counterbalanced across participants, and the assignment of the three maps into each condition was randomly decided by computer for each participant.

During the practice phase, the restudy procedure was identical to that in Experiments 1 and 2. The procedure in the forward practice condition changed slightly. Specifically, participants gradually moved the cursor from the starting point to the destination. When participants pressed a button to move the cursor, a passing-by trajectory from the current position to the next correct position was concurrently presented on the screen (see Figure 4). In the backward practice condition, participants set off at the destination of the initially learned map and needed to move the cursor from the destination back to the starting point, with a passing-by trajectory shown on the screen. After the practice phase, participants were dismissed and returned to the lab 24 h later to complete a final test (i.e., a backward navigation test), which was identical to that in Experiment 2. Experiment 3 omitted the final test on peripheral information.

### 6.2. Results and Discussion

**Figure 5:**
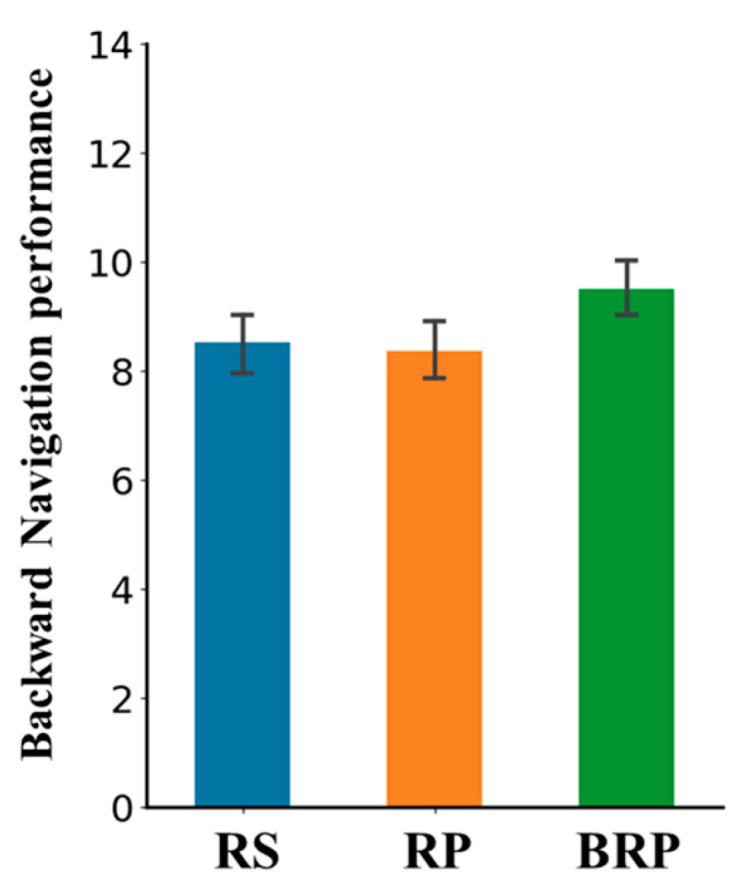
Results of Experiment 3. *Note.* RS = restudy; RP = retrieval practice; BRP = backward retrieval practice. Error bars indicate 95% confidence interval.

A repeated-measures ANOVA showed a main effect of study strategy on final test performance (i.e., backward navigation performance); *F*(2,110) = 12.16, *p* < .001, η_p_^2^ = 0.18, *BF*_10_ > 1000. As shown in Figure 5, a post hoc analysis indicated that there was minimal difference in backward navigation performance between the restudy and forward practice conditions; difference = 0.14 95% CI = [−0.230, 0.372], *t*(55) = 0.58, *p* = .57, *d* = 0.07, *BF*_10_ = 0.17. However, performance in the backward practice condition was substantially better than that in the restudy condition, difference = 0.982 95% CI = [0.167, 0.809], *t*(55) = 3.954, *p* < .001, *d* = 0.488, *BF*_10_ = 143.55, and also better than that in the forward practice condition, difference = 1.125 95% CI = [0.232, 0.886], *t*(55) = 4.530, *p* < .001, *d* = 0.559, *BF*_10_ = 468.17.

Experiment 3 showed that providing concurrent trajectory enhanced visuospatial representation of the route and eliminated the negative effect of forward navigation test on backward navigation performance. However, it did not overturn the effect. Furthermore, Experiment 3 demonstrated that the practice test of backward navigation substantially enhanced backward navigation performance by comparison with restudying, re-confirming the enhancing effect of testing.

To further understand the mechanisms underlying the negative testing effect on backward navigation, we probed the serial position effect, examining participant’s performance at each of the 16 crosses in our study. Anticipating the impact of primacy and recency effects, a pattern of memory function in which performance would initially deteriorate, then improve across the cross indices is hypothesized. Consequently, the relationship between navigation accuracy and cross indices (1–16) should be represented by a quadratic function. Data from Experiment 1 (frontward navigation) and Experiment 2 (backward navigation) were extracted for analysis comparison. Quadratic regressions were conducted with navigation accuracy (i.e., the proportion of participants who successfully responded at a given cross) as the dependent variable and cross index as the independent variable. Table 1 shows linear coefficients and quadratic coefficients for the regression of four conditions (i.e., forward/backward × restudy/testing). In the quadratic regression, the coefficients of the linear coefficients (β_1_) and the quadratic coefficients (β_2_), respectively, determine the magnitude of the decrease and increase trends of the dependent variable around the lowest point.

**Figure 6:**
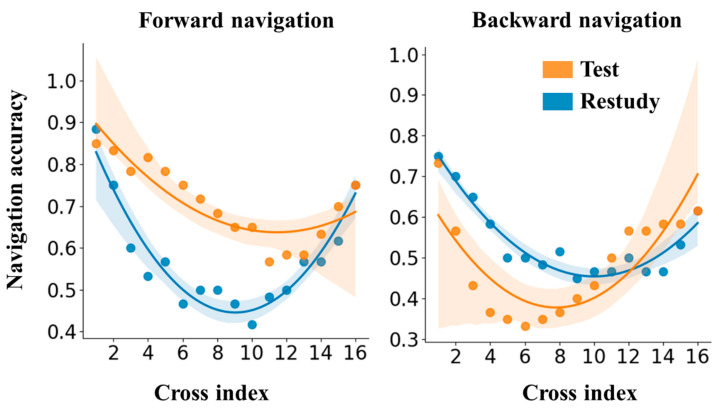
Quadratic regressions between cross index and navigation accuracy for the forward and backward navigation.

Figure 6 depicts that retrieval practice under the forward navigation condition offers a protection for items distant from both the start and endpoint, ensuring that the memory decline of intermediate items is not as substantial as in the restudy condition. This outcome aligns with the episodic context account, which presumes retrieval practice established additional temporal cues to facilitate memory (Lehman et al. 2014). Conversely, during the final test of backward navigation, retrieval practice resulted in a more extensive decline for the intermediate crosses. To statistically affirm this, we drew comparisons between β1 (the linear coefficient) and β2 (the quadratic coefficient) utilizing Clogg et al.’s (1995) technique for comparing coefficients of two independent regressions. As exhibited in Table 2, under the forward navigation condition, restudying resulted in larger β1 and β2 in contrast to testing, indicating a less severe decline in the memory of intermediate items. However, no significant difference was observed in these two coefficients in the backward navigation condition.

The above results provide a nuanced examination of how testing effect was overturned in the context of backward navigation. As the episodic context account articulates, enhancing temporal cues is vital for elucidating the testing effect, leading to a “protection” effect for intermediate items under forward navigation conditions. However, in backward navigation conditions, the temporal cues established during retrieval practice are deconstructed, voiding the “protection” effect for these intermediate items. Notably, previous research has demonstrated that context mismatch can wipe out the testing effect (Whiffen and Karpicke 2017). It would be worthwhile for future research to investigate whether the testing effect is weakened when final recall necessitates a reversed order as compared to retrieval practice during sequential material learning.

## 7. General Discussion

In the present study, we demonstrated the testing effect for map route learning, illustrating that retrieval practice facilitates memory retention for both the route and the peripheral information along it. Together with previous studies, the testing effect was found to be evident across a spectrum of map learning tasks, including learning item position (Carpenter and Pashler 2007), learning the name of different locations (Rohrer et al. 2010), learning the inter-item orientations (Carpenter and Kelly 2012), and learning the route between two points (the present study). Nonetheless, we observed a noteworthy exception: the testing effect was counteracted when participants were required to recall the route in reverse (i.e., backward navigation). This outcome presents an exceptionally rare instance where restudy outperforms retrieval practice in the realm of testing effect investigations (for reviews, see Rowland 2014; Yang et al. 2021).

### 7.1. The Positive Effect of Retrieval Practice on Map Learning (Experiment 1)

The transfer of the testing effect has been confirmed irrespective of whether the testing format and knowledge domain of the final test differed from those of the retrieval practice or not. This benefit has also been supported by previous investigations of the testing effect on map learning (Carpenter and Kelly 2012; Carpenter and Pashler 2007; Rohrer et al. 2010). Different from the previous studies, the present study demonstrates that testing can enhance the memory of the information that is not directly involved in the retrieval practice session.

Most previous investigations have measured transfer learning through the paradigm of reverse operations (e.g., Carpenter and Kelly 2012: retrieval practice involves identifying point B at A, while the final test entails recognizing point A at B; Rohrer et al. 2010: retrieval practice requires recalling a city’s position and the final test requires recalling a position’s city name). In these cases, although the testing format changed, the transfer task did not involve extra information that was not covered by retrieval practice. Thus, a pivotal insight from this study is that retrieval practice bolsters recall of not only the tested information but also the untested facts, which were presented concurrently during the encoding phase.

That recalling the route enhances the memory of peripheral information may be attributed to the concept that retrieval practice induces a deeper integration of semantic and contextual meaning (Endres and Renkl 2015; Lehman et al. 2014). Essentially, this suggests that participants tend to consolidate information related to the target memory to facilitate recall. According to previous investigations with verbal material, semantic elaboration is viewed as a crucial mechanism underpinning the testing effect (Carpenter 2009). More specifically, retrieval practice can activate semantic information related to the target, thus fostering an in-depth semantic processing. For instance, participants might create an associative word to aid memory of word pairs (e.g., basket-egg-breakfast for basket-breakfast). In the context of the current study, this semantic elaboration could also play a role in giving retrieval practice an edge in map learning. At each cross where a decision must be taken, participants may actively associate the route with peripheral information (e.g., KFC-left). This was consistent with the observation that the more the memory of peripheral information is amplified, the larger the testing effect upon the route. However, elaboration is evidently not the sole mechanism that underlies the testing effect on map learning, as the testing effect, albeit decreasing, persisted in the no-OR condition. This underscores a non-semantic interpretation of the testing effect. For example, the episodic explanation (Karpicke et al. 2014) posits that the act of retrieval practice can assimilate the concurrent contexts and so increase the abundance of retrieval cues in the final test.

Despite substantial research indicating that retrieval practice enhances memory of non-practiced information (i.e., test-enhanced transfer learning), it remains unclear if the improvement of untested information is a cause or a result of the enhancement of tested information. The present study addressed this issue by showing that an improved memory was observed for both the route and its peripheral information. Interestingly, the scale of the testing effect on route memory correlates positively with the scale of the testing effect on peripheral information. Experiment 1 discounted the possibility that the superior peripheral information memory was just an outcome of better route memory by presenting evidence that, when peripheral information was eliminated, the testing effect was diminished. If the enhancement of peripheral information was a by-product of route improvement, removing RO should not influence the scale of the testing effect. Thus, the study supports the idea that the more active use of peripheral information is responsible for the beneficial effect of retrieval practice on map learning.

### 7.2. Negative Effects of Retrieval Practice on Map Learning (Experiments 2 and 3)

While the transfer of the testing effect has been widely validated, this study reveals that, in the context of map learning, retrieval practice is not always the best fit for all transfer tasks. In fact, it appears that retrieval practice hinders backward navigation memory compared to restudying. A potential explanation is that, although retrieval practice enhances semantic elaboration, it might simultaneously degrade visuospatial representation of the route (Meissner and Brigham 2001; Schooler and Engstler-Schooler 1990). This degradation is particularly noteworthy as successful backward navigation may fundamentally rely on mental rotation of this representation. However, in the restudy condition, the route is presented in an intact manner, leading to a superior visuospatial representation. Although Experiments 1 and 2 consistently demonstrated enhanced memorization of the route’s peripheral information via retrieval practice, there tends to be no relation between the testing effects on memory of the route and peripheral information in the backward navigation condition. This may be attributed to the failure of semantic elaboration in aiding recall, as backward navigation seems to deconstruct the association between the route and peripheral information. To elaborate, directional indicators such as “KFC-left” would need to be flipped to “KFC-right” in the context of backward navigation. The result can be linked with transfer-appropriate processing theory (Morris et al. 1977), which posits that the effectiveness of retrieval practice depends on the alignment between the cognitive processes engaged during practice and those required during the final test. In forward navigation, the sequential decision-making processes during retrieval practice closely match those needed in the final test, leading to enhanced performance. However, in backward navigation, the cognitive processes differ significantly, as they require mental rotation and reorganization of the route, which are not adequately supported by retrieval practice. This misalignment may explain why retrieval practice impaired backward navigation performance.

Experiment 3 provided validation for this assumption; the introduction of a simultaneous trajectory during the retrieval practice session eliminated the negative impact of retrieval practice. This suggests that visualization of the trajectory contributed to a better visuospatial representation of the route. However, this adjustment merely counteracted the deficit of retrieval practice but did not bring a positive testing effect. The reason behind this is quite clear: although including a trajectory fortified visuospatial representation, the act of incrementally presenting the route still falls short by comparison with presenting the complete route at once, as in the restudy condition. In other words, progressive presentation fails to create as rich and detailed a visuospatial representation as viewing the intact route does. According to episodic context theory (Lehman et al. 2014; Whiffen and Karpicke 2017), retrieval practice strengthens the episodic context associated with the learned material, which aids recall in tasks that rely on the same context. In forward navigation, the episodic context established during retrieval practice aligns with the final test, facilitating recall. However, in backward navigation, the episodic context is disrupted, as the task requires a reversal of the learned sequence, leading to poorer performance. This disruption of context may further explain the negative testing effect observed in backward navigation.

In conclusion, forward navigation and backward navigation involve distinct cognitive processes and exhibit different reactions to retrieval practice and restudy. Various theories discuss the trade-off between item-specific processing and processing of inter-item relationships (McDaniel and Bugg 2008; Zhao et al. 2023) or the trade-off between the detailed visual feature of overall recognition performance, also known as verbal overshadowing (Meissner and Brigham 2001; Schooler and Engstler-Schooler 1990). Our study affirms a similar trade-off; enhancing the pictorial details of a route can dismantle visuospatial representation. This insight can pave the way for future research. The trade-off may not be limited to map-learning scenarios but could extend to other visuospatial tasks. Broadly speaking, if retrieval practice concentrates on semantic elaboration of segmented features of an object, might it undermine memory representation of the entire object? We encourage future research to thoroughly investigate this question.

## 8. Limitations and Practical Implications

A limitation of the present study is that the maps were learned, practiced, and used (i.e., tested) in a 2D fashion across the experiment. However, a more common situation is that one learns the route on a 2D map but uses this 2D map to guide the direction in the 3D real world, although studies have demonstrated that the performance of a 2D navigation can predict the performance of 3D navigation (Remazeilles et al. 2006). Another limitation is that, in our experimental materials, roads are only horizontal or vertical and the size of each block was equal. However, a real map can be much more complex. Regardless of this, we hypothesize that the effects reported by the present study will persist or even be enlarged with more complex maps, as, in a real map, the abundance of an object is increased and this might enlarge the effect of semantic elaboration.

Another limitation of the present study is the scope of the retrieval practice design. While our findings demonstrate the effectiveness of retrieval practice for forward navigation, the negative effect observed in backward navigation suggests that the benefits of retrieval practice may be task-specific and dependent on the alignment between the practice format and the final test. It is possible that the narrow focus on forward navigation during retrieval practice contributed to the observed encoding specificity, making it difficult for participants to adapt to the reversed task. Future studies could explore whether broadening the scope of retrieval practice—for example, by incorporating both forward and backward navigation during training—could mitigate this negative effect. This approach would be analogous to foreign language learning, where testing oneself in both directions (e.g., from the foreign language to the native language and vice versa) is essential for robust learning. Such a design could provide insights into how retrieval practice can be optimized to enhance flexibility and adaptability across different task demands.

Several theoretical possibilities may account for the negative effect of retrieval practice on backward navigation. First, retrieval practice may strengthen sequential associations between elements in a forward direction (e.g., A→B→C), facilitating recall of the next step but potentially impairing recall of the reverse sequence (C→B→A). This aligns with the sequential association theory, which posits that retrieval practice enhances forward linkages at the expense of backward linkages (Anderson 1983). Second, retrieval practice may lead to reduced contextual encoding, as learners focus on retrieving isolated forward links rather than encoding the broader spatial or relational structure of the route. This is consistent with the item-specific processing hypothesis, which suggests that retrieval practice prioritizes item-specific details over relational or contextual information (McDaniel and Bugg 2008). Third, retrieval practice could induce proactive interference, where the strengthened forward associations disrupt the recall of previous locations, particularly in tasks requiring mental rotation or reorganization of the route (Bjork 1994). Finally, differences in cognitive load may play a role; forward navigation may rely more on procedural or habitual memory, which is effectively supported by retrieval practice, whereas backward navigation may require more conscious effort and mental manipulation, which retrieval practice does not adequately facilitate (Paas et al. 2003). It is worth justifying these possibilities, though they are conceptually consistent with the explanations proposed in the present study.

The current findings suggest that, in a map learning situation, the benefit of retrieval practice is task-specific. That is, the testing effect on map learning critically relies on the consistency between retrieval practice and final recall. Thus, if one wants to benefit from retrieval practice in map learning, two suggestions should be considered: (a) keep the task consistent between retrieval practice and the final test; (b) set multiple tasks during retrieval practice (e.g., forward navigation + backward navigation).

## 9. Conclusions

Retrieval practice is a double-edged sword for map learning. On the one hand, by activating semantic integration of peripheral information, a testing effect emerged. On the other hand, retrieval practice impairs the integrity of visuospatial representation of the route and so leads to worse performance in backward navigation, which requires a mental rotation of the initially learned route. The present study addressed a pervasive trade-off in memory literature that deep processing of details or segmentary information impaired memory of the whole representation.

## Figures and Tables

**Table 1:** Quadratic regression result of Experiment 3.

Condition		Coefficient	SE	*t*	*p*
Forward-Restudy	Intercept	55.78	2.05	27.14	<.001
	*β*_1_	−6.41	0.56	−11.52	<.001
	*β*_2_	0.35	0.03	11.13	<.001
Forward-Test	Intercept	56.90	2.37	24.01	<.001
	*β*_1_	−3.26	0.64	−5.08	<.001
	*β*_2_	0.14	0.04	3.87	.002
Backward-Restudy	Intercept	49.26	1.41	34.87	<.001
	*β*_1_	−4.39	0.38	−11.49	<.001
	*β*_2_	0.22	0.02	10.03	<.001
Backward-Test	Intercept	40.59	3.69	11.00	<.001
	*β*_1_	−4.58	1.00	−4.58	<.001
	*β*_2_	0.29	0.06	5.13	.002

**Table 2:** Coefficient comparisons of regressions in Experiment 3.

	z	*p*
β_1_: Restudy vs. Testing in FN	3.70	<.001
β_2_: Restudy vs. Testing in FN	4.19	<.001
β_1_: Restudy vs. Testing in BN	1.11	.26
β_2_: Restudy vs. Testing in BN	−0.18	.86

*Note.* FN = forward navigation; BN = backward navigation.

## Data Availability

All the data are available from the OSF link: https://osf.io/gy42a/?view_only=dc480cb3288c4e8a852d42f07c66c49f (accessed on 6 April 2025).

## References

[B1-jintelligence-13-00049] Anderson John R. (1983). A spreading activation theory of memory. Journal of Verbal Learning and Verbal Behavior.

[B2-jintelligence-13-00049] Bjork Robert A. (1994). Memory and metamemory considerations in the training of human beings. Metacognition: Knowing about knowing.

[B3-jintelligence-13-00049] Carpenter Shana K. (2009). Cue strength as a moderator of the testing effect: The benefits of elaborative retrieval. Journal of Experimental Psychology: Learning, Memory, and Cognition.

[B4-jintelligence-13-00049] Carpenter Shana K., DeLosh Edward L. (2005). Application of the testing and spacing effects to name learning. Applied Cognitive Psychology: The Official Journal of the Society for Applied Research in Memory and Cognition.

[B5-jintelligence-13-00049] Carpenter Shana K., Pashler Harold (2007). Testing beyond words: Using tests to enhance visuospatial map learning. Psychonomic Bulletin & Review.

[B6-jintelligence-13-00049] Carpenter Steven K., Kelly Jonathan W. (2012). Tests enhance retention and transfer of spatial learning. Psychonomic Bulletin & Review.

[B7-jintelligence-13-00049] Clogg Clifford C., Petkova Elizabeth, Haritou Alex (1995). Statistical methods for comparing regression coefficients between models. American Journal of Sociology.

[B8-jintelligence-13-00049] Endres Tobias, Renkl Alexander (2015). Mechanisms behind the testing effect: An empirical investigation of retrieval practice in meaningful learning. Frontiers in Psychology.

[B9-jintelligence-13-00049] Kang Sean H. (2010). Enhancing visuospatial learning: The benefit of retrieval practice. Memory & Cognition.

[B10-jintelligence-13-00049] Karpicke Jeffrey D., Lehman Melissa, Aue William R. (2014). Retrieval-based learning: An episodic context account. Psychology of Learning and Motivation.

[B11-jintelligence-13-00049] Lehman Melissa, Smith Megan A., Karpicke Jeffrey D. (2014). Toward an episodic context account of retrieval-based learning: Dissociating retrieval practice and elaboration. Journal of Experimental Psychology: Learning, Memory, and Cognition.

[B12-jintelligence-13-00049] McDaniel Mark A., Bugg Julie M. (2008). Instability in memory phenomena: A common puzzle and a unifying explanation. Psychonomic Bulletin & Review.

[B13-jintelligence-13-00049] McDaniel Mark A., Anderson Janis L., Derbish Mary H., Morrisette Nova (2007). Testing the testing effect in the classroom. European Journal of Cognitive Psychology.

[B14-jintelligence-13-00049] Meissner Christian A., Brigham John C. (2001). A meta-analysis of the verbal overshadowing effect in face identification. Applied Cognitive Psychology: The Official Journal of the Society for Applied Research in Memory and Cognition.

[B15-jintelligence-13-00049] Montoya Amanda K. (2019). Moderation analysis in two-instance repeated measures designs: Probing methods and multiple moderator models. Behavior Research Methods.

[B16-jintelligence-13-00049] Morris C. Donald, Bransford John D., Franks Jeffery J. (1977). Levels of processing versus transfer appropriate processing. Journal of Verbal Learning and Verbal Behavior.

[B17-jintelligence-13-00049] Paas Fred, Renkl Alexander, Sweller John (2003). Cognitive load theory and instructional design: Recent developments. Educational Psychologist.

[B18-jintelligence-13-00049] Peirce Jonathan W. (2007). PsychoPy—Psychophysics software in Python. Journal of Neuroscience Methods.

[B19-jintelligence-13-00049] Remazeilles Anthony, Chaumette François, Gros Patrick (2006). 3D navigation based on a visual memory. Paper presented at IEEE International Conference on Robotics and Automation.

[B20-jintelligence-13-00049] Roediger Henry L., Karpicke Jeffrey D. (2006). Test-enhanced learning: Taking memory tests improves long-term retention. Psychological Science.

[B21-jintelligence-13-00049] Rohrer Doug, Taylor Kelli, Sholar Brandon (2010). Tests enhance the transfer of learning. Journal of Experimental Psychology: Learning, Memory, and Cognition.

[B22-jintelligence-13-00049] Rowland Catherine A. (2014). The effect of testing versus restudy on retention: A meta-analytic review of the testing effect. Psychological Bulletin.

[B23-jintelligence-13-00049] Schooler Jonathan W., Engstler-Schooler Tonya Y. (1990). Verbal overshadowing of visual memories: Some things are better left unsaid. Cognitive Psychology.

[B24-jintelligence-13-00049] Shanks David R., Don Hilary J., Boustani Shaun, Yang Chunliang (2023). Test-enhanced learning. Oxford Research Encyclopedia of Psychology.

[B25-jintelligence-13-00049] Whiffen Joshua W., Karpicke Jeffrey D. (2017). The role of episodic context in retrieval practice effects. Journal of Experimental Psychology: Learning, Memory, and Cognition.

[B26-jintelligence-13-00049] Yang Chunliang, Luo Liang, Vadillo Miguel A., Yu Rongjun, Shanks David R. (2021). Testing (quizzing) boosts classroom learning: A systematic and meta-analytic review. Psychological Bulletin.

[B27-jintelligence-13-00049] Zhao Wenbo, Li Jiaojiao, Shanks David R., Li Baike, Hu Xiao, Yang Chunliang, Luo Liang (2023). Metamemory judgments have dissociable reactivity effects on item and interitem relational memory. Journal of Experimental Psychology: Learning, Memory, and Cognition.

